# Bis(2-imino­methyl-5-methoxy­phenolato)nickel(II)

**DOI:** 10.1107/S1600536809039233

**Published:** 2009-10-03

**Authors:** Chunbao Tang

**Affiliations:** a Department of Chemistry, Jiaying University, Meizhou 514015, People’s Republic of China

## Abstract

The title compound, [Ni(C_8_H_8_NO_2_)_2_], is a centrosymmetric mononuclear nickel(II) complex. The Ni^II^ ion, lying on an inversion centre, is four-coordinated in a square-planar geometry by two phenolate O and two imine N atoms from two symmetry-related 2-imino­methyl-5-methoxy­phenolate ligands. In the crystal, mol­ecules are linked into corrugated layers parallel to (100) by N—H⋯O hydrogen bonds.

## Related literature

For related structures, see: Angulo *et al.* (2001 ▶); Dey *et al.* (2004 ▶); Edison *et al.* (2004 ▶); Ramadevi *et al.* (2005 ▶); Suh *et al.* (1996 ▶); Tang (2009 ▶); Kamenar *et al.* (1990 ▶); Costes *et al.* (1994 ▶).
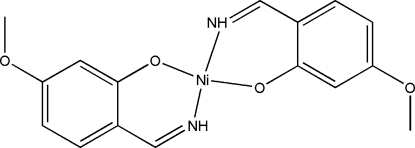


## Experimental

### 

#### Crystal data

[Ni(C_8_H_8_NO_2_)_2_]
*M* *_r_* = 359.02
Orthorhombic, 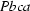

*a* = 7.5704 (16) Å
*b* = 11.331 (2) Å
*c* = 17.227 (4) Å
*V* = 1477.7 (5) Å^3^
*Z* = 4
Mo *K*α radiation
μ = 1.34 mm^−1^
*T* = 298 K
0.18 × 0.17 × 0.17 mm

#### Data collection

Bruker SMART CCD area-detector diffractometer
Absorption correction: multi-scan (*SADABS*; Sheldrick, 1996 ▶) *T*_min_ = 0.795, *T*_max_ = 0.805
7939 measured reflections
1620 independent reflections
1122 reflections with *I* > 2σ(*I*)
*R*_int_ = 0.028

#### Refinement

*R*[*F* ^2^ > 2σ(*F* ^2^)] = 0.028
*wR*(*F* ^2^) = 0.083
*S* = 1.01
1620 reflections
110 parameters
1 restraint
H atoms treated by a mixture of independent and constrained refinement
Δρ_max_ = 0.28 e Å^−3^
Δρ_min_ = −0.29 e Å^−3^

### 

Data collection: *SMART* (Bruker, 2002 ▶); cell refinement: *SAINT* (Bruker, 2002 ▶); data reduction: *SAINT*; program(s) used to solve structure: *SHELXS97* (Sheldrick, 2008 ▶); program(s) used to refine structure: *SHELXL97* (Sheldrick, 2008 ▶); molecular graphics: *SHELXTL* (Sheldrick, 2008 ▶); software used to prepare material for publication: *SHELXL97*.

## Supplementary Material

Crystal structure: contains datablocks global, I. DOI: 10.1107/S1600536809039233/ci2923sup1.cif

Structure factors: contains datablocks I. DOI: 10.1107/S1600536809039233/ci2923Isup2.hkl

Additional supplementary materials:  crystallographic information; 3D view; checkCIF report

## Figures and Tables

**Table:** 

Ni1—O1	1.8411 (16)
Ni1—N1	1.8529 (18)

**Table:** 

O1^i^—Ni1—O1	180
O1—Ni1—N1^i^	86.08 (6)
O1—Ni1—N1	93.92 (6)
N1^i^—Ni1—N1	180

Symmetry code: (i) 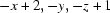.

**Table 2:** Hydrogen-bond geometry (Å, °)

*D*—H⋯*A*	*D*—H	H⋯*A*	*D*⋯*A*	*D*—H⋯*A*
N1—H1⋯O2^ii^	0.90 (1)	2.391 (18)	3.166 (2)	144 (2)

Symmetry code: (ii) 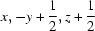.

**Fig. 1.**
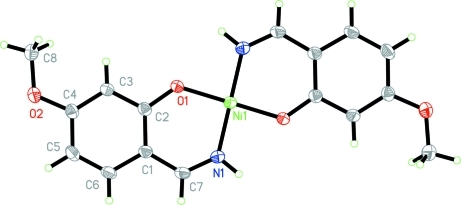
The molecular structure of the title complex. Displacement ellipsoids are drawn at the 30% probability level. Unlabelled atoms are at the symmetry position (2-x, -y, 1-z).

**Fig. 2.**
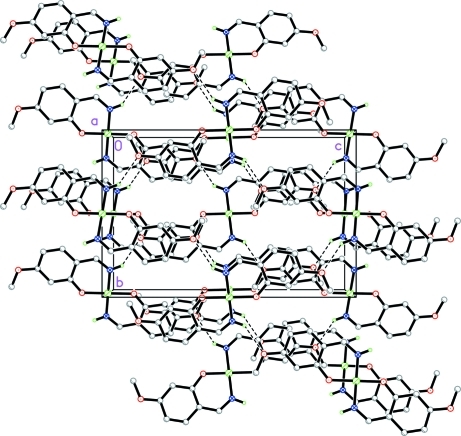
Packing diagram, viewed along the a axis. Hydrogen bonds are shown as dashed lines.

## supplementary crystallographic information

### Comment

Nickel(II) complexes play an important role in both bioinorganic chemistry and coordination chemistry (Suh *et al.*, 1996; Dey *et al.*, 2004; Angulo *et al.*, 2001; Ramadevi *et al.*, 2005; Edison *et al.*, 2004). Recently, the author has reported a nickel(II) complex (Tang, 2009). As a continuation of this work, the title mononuclear nickel(II) complex (Fig. 1), is reported in this paper.

The title complex is a centrosymmetric mononuclear nickel(II) complex. The Ni^II^ ion, lying on the inversion centre, is four-coordinated in a square-planar geometry, with two phenolate O and two imine N atoms from two 2-(iminomethyl)-5-methoxyphenolate ligands. The coordination bond lengths (Table 1) are comparable to those observed in related complexes (Kamenar *et al.*, 1990; Costes *et al.*, 1994).

In the crytal structure, molecules are linked through intermolecular N—H···O hydrogen bonds (Table 2), forming zigzag layers parallel to the (100) [Fig.2].

### Experimental

4-Methoxy-2-hydroxybenzaldehyde (0.2 mmol, 30.5 mg) and nickel(II) nitrate hexahydrate (0.1 mmol, 29.1 mg) were mixed in a methanol solution (20 ml) which contains small quantity of ammonia. The mixture was stirred at room temperature for 30 min to give a red solution. The solution was allowed to stand in air for 8 d, yielding red block-shaped crystals of the title complex. The absorption band indicative of the C═N double bond formation in the IR spectrum of the complex is at 1617 cm^-1^.

### Refinement

Atom H1 was located in a difference Fourier map and refined isotropically, with N-H distance restrained to 0.90 (1) Å and U_iso_ set at 0.08 Å^2^. Other H atoms were constrained to ideal geometries, with C-H = 0.93–0.96 Å and *U*_iso_(H) = 1.2*U*_eq_(C) and 1.5*U*_eq_(C8).

### Crystal data

**Table:**

[Ni(C_8_H_8_NO_2_)_2_]	*F*(000) = 744
*M**_r_* = 359.02	*D*_x_ = 1.614 Mg m^−^^3^
Orthorhombic, *P**b**c**a*	Mo *K*α radiation, λ = 0.71073 Å
Hall symbol: -P 2ac 2ab	Cell parameters from 1894 reflections
*a* = 7.5704 (16) Å	θ = 2.3–26.2°
*b* = 11.331 (2) Å	µ = 1.34 mm^−^^1^
*c* = 17.227 (4) Å	*T* = 298 K
*V* = 1477.7 (5) Å^3^	Block, red
*Z* = 4	0.18 × 0.17 × 0.17 mm

### Data collection

**Table:**

Bruker SMART CCD area-detector diffractometer	1620 independent reflections
Radiation source: fine-focus sealed tube	1122 reflections with *I* > 2σ(*I*)
graphite	*R*_int_ = 0.028
ω scans	θ_max_ = 27.0°, θ_min_ = 2.4°
Absorption correction: multi-scan (*SADABS*; Sheldrick, 1996)	*h* = −9→6
*T*_min_ = 0.795, *T*_max_ = 0.805	*k* = −11→14
7939 measured reflections	*l* = −21→22

### Refinement

**Table:**

Refinement on *F*^2^	Primary atom site location: structure-invariant direct methods
Least-squares matrix: full	Secondary atom site location: difference Fourier map
*R*[*F*^2^ > 2σ(*F*^2^)] = 0.028	Hydrogen site location: inferred from neighbouring sites
*wR*(*F*^2^) = 0.083	H atoms treated by a mixture of independent and constrained refinement
*S* = 1.01	*w* = 1/[σ^2^(*F*_o_^2^) + (0.0414*P*)^2^ + 0.384*P*] where *P* = (*F*_o_^2^ + 2*F*_c_^2^)/3
1620 reflections	(Δ/σ)_max_ = 0.001
110 parameters	Δρ_max_ = 0.28 e Å^−^^3^
1 restraint	Δρ_min_ = −0.29 e Å^−^^3^

### Special details

**Table:**

Geometry. All esds (except the esd in the dihedral angle between two l.s. planes) are estimated using the full covariance matrix. The cell esds are taken into account individually in the estimation of esds in distances, angles and torsion angles; correlations between esds in cell parameters are only used when they are defined by crystal symmetry. An approximate (isotropic) treatment of cell esds is used for estimating esds involving l.s. planes.
Refinement. Refinement of F^2^ against ALL reflections. The weighted R-factor wR and goodness of fit S are based on F^2^, conventional R-factors R are based on F, with F set to zero for negative F^2^. The threshold expression of F^2^ > 2sigma(F^2^) is used only for calculating R-factors(gt) etc. and is not relevant to the choice of reflections for refinement. R-factors based on F^2^ are statistically about twice as large as those based on F, and R- factors based on ALL data will be even larger.

### Fractional atomic coordinates and isotropic or equivalent isotropic displacement parameters (Å^2^)

**Table:**

	*x*	*y*	*z*	*U*_iso_*/*U*_eq_	
Ni1	1.0000	0.0000	0.5000	0.03483 (14)	
O1	0.98642 (18)	0.00398 (11)	0.39332 (9)	0.0407 (4)	
O2	0.8757 (2)	0.14990 (13)	0.13928 (8)	0.0486 (4)	
N1	0.9039 (3)	0.14925 (16)	0.51213 (9)	0.0422 (4)	
C1	0.8581 (3)	0.19740 (17)	0.37798 (11)	0.0366 (5)	
C2	0.9275 (3)	0.08998 (17)	0.34895 (11)	0.0352 (4)	
C3	0.9336 (3)	0.07278 (17)	0.26800 (11)	0.0369 (5)	
H3	0.9783	0.0026	0.2480	0.044*	
C4	0.8738 (3)	0.15895 (18)	0.21830 (11)	0.0380 (5)	
C5	0.8038 (3)	0.26481 (18)	0.24643 (12)	0.0457 (5)	
H5	0.7634	0.3224	0.2123	0.055*	
C6	0.7956 (3)	0.28230 (19)	0.32452 (12)	0.0426 (5)	
H6	0.7473	0.3522	0.3433	0.051*	
C7	0.8489 (3)	0.22017 (18)	0.45884 (12)	0.0423 (5)	
H7	0.7997	0.2915	0.4746	0.051*	
C8	0.9403 (4)	0.0435 (2)	0.10637 (13)	0.0547 (6)	
H8A	1.0625	0.0338	0.1198	0.082*	
H8B	0.9286	0.0467	0.0509	0.082*	
H8C	0.8736	−0.0220	0.1261	0.082*	
H1	0.897 (3)	0.176 (2)	0.5612 (8)	0.080*	

### Atomic displacement parameters (Å^2^)

**Table:**

	*U*^11^	*U*^22^	*U*^33^	*U*^12^	*U*^13^	*U*^23^
Ni1	0.0469 (2)	0.0262 (2)	0.0314 (2)	0.00123 (15)	−0.00135 (15)	−0.00138 (14)
O1	0.0622 (10)	0.0268 (8)	0.0330 (7)	0.0072 (6)	−0.0022 (6)	0.0002 (5)
O2	0.0658 (10)	0.0432 (9)	0.0367 (8)	0.0046 (7)	−0.0027 (7)	0.0072 (7)
N1	0.0578 (12)	0.0320 (10)	0.0367 (10)	0.0037 (9)	−0.0006 (8)	−0.0044 (7)
C1	0.0413 (11)	0.0290 (10)	0.0395 (11)	−0.0004 (8)	−0.0032 (8)	−0.0019 (9)
C2	0.0384 (11)	0.0289 (11)	0.0382 (11)	−0.0038 (9)	−0.0029 (8)	0.0015 (8)
C3	0.0442 (11)	0.0284 (10)	0.0381 (11)	0.0001 (9)	0.0005 (9)	−0.0001 (8)
C4	0.0402 (12)	0.0361 (11)	0.0378 (11)	−0.0045 (9)	−0.0036 (8)	0.0045 (9)
C5	0.0532 (12)	0.0351 (12)	0.0488 (13)	0.0023 (10)	−0.0072 (10)	0.0107 (10)
C6	0.0499 (13)	0.0279 (11)	0.0500 (13)	0.0058 (9)	−0.0032 (10)	0.0007 (10)
C7	0.0516 (14)	0.0281 (11)	0.0472 (13)	0.0038 (10)	−0.0018 (10)	−0.0050 (9)
C8	0.0743 (16)	0.0515 (14)	0.0382 (12)	0.0055 (13)	−0.0010 (11)	0.0029 (11)

### Geometric parameters (Å, °)

**Table:**

Ni1—O1^i^	1.8411 (16)	C2—C3	1.409 (3)
Ni1—O1	1.8411 (16)	C3—C4	1.375 (3)
Ni1—N1^i^	1.8529 (18)	C3—H3	0.93
Ni1—N1	1.8529 (18)	C4—C5	1.398 (3)
O1—C2	1.316 (2)	C5—C6	1.361 (3)
O2—C4	1.365 (2)	C5—H5	0.93
O2—C8	1.419 (3)	C6—H6	0.93
N1—C7	1.289 (3)	C7—H7	0.93
N1—H1	0.901 (10)	C8—H8A	0.96
C1—C6	1.413 (3)	C8—H8B	0.96
C1—C2	1.417 (3)	C8—H8C	0.96
C1—C7	1.418 (3)		
			
O1^i^—Ni1—O1	180	C2—C3—H3	119.8
O1^i^—Ni1—N1^i^	93.92 (6)	O2—C4—C3	124.35 (19)
O1—Ni1—N1^i^	86.08 (6)	O2—C4—C5	114.44 (18)
O1^i^—Ni1—N1	86.08 (6)	C3—C4—C5	121.21 (19)
O1—Ni1—N1	93.92 (6)	C6—C5—C4	119.00 (19)
N1^i^—Ni1—N1	180	C6—C5—H5	120.5
C2—O1—Ni1	128.08 (13)	C4—C5—H5	120.5
C4—O2—C8	117.75 (16)	C5—C6—C1	121.97 (19)
C7—N1—Ni1	127.97 (15)	C5—C6—H6	119.0
C7—N1—H1	116.0 (17)	C1—C6—H6	119.0
Ni1—N1—H1	116.0 (17)	N1—C7—C1	124.78 (19)
C6—C1—C2	118.63 (18)	N1—C7—H7	117.6
C6—C1—C7	119.98 (18)	C1—C7—H7	117.6
C2—C1—C7	121.40 (18)	O2—C8—H8A	109.5
O1—C2—C3	117.48 (18)	O2—C8—H8B	109.5
O1—C2—C1	123.82 (17)	H8A—C8—H8B	109.5
C3—C2—C1	118.69 (18)	O2—C8—H8C	109.5
C4—C3—C2	120.50 (19)	H8A—C8—H8C	109.5
C4—C3—H3	119.8	H8B—C8—H8C	109.5

Symmetry codes: (i) −*x*+2, −*y*, −*z*+1.

### Hydrogen-bond geometry (Å, °)

**Table:**

*D*—H···*A*	*D*—H	H···*A*	*D*···*A*	*D*—H···*A*
N1—H1···O2^ii^	0.90 (1)	2.39 (2)	3.166 (2)	144 (2)

Symmetry codes: (ii) *x*, −*y*+1/2, *z*+1/2.
